# Convective heat transfer coefficient relating to evaluation of thermal environment of infant

**DOI:** 10.1016/j.heliyon.2022.e12076

**Published:** 2022-12-05

**Authors:** Yoshihito Kurazumi, Kenta Fukagawa, Tomonori Sakoi, Ken Yamashita, Akie Naito, Motoe Imai, Yoshiaki Yamato, Emi Kondo, Tadahiro Tsuchikawa

**Affiliations:** a School of Life Studies, Sugiyama Jogakuen University, Nagoya, Japan; b Department of Environmental Science and Technology, Meijo University, Japan; c Department of Advanced Textile and Kansei Engineering, Shinshu University, Ueda, Japan; d Institute for Global Leadership, Ochanomizu University, Tokyo, Japan; e Graduate School of Life Studies, Sugiyama Jogakuen University, Nagoya, Japan; f Department of Architecture and Structural Engineering, Kure National College of Technology, Kure, Japan; g Department of Comprehensive Engineering, Kindai University Technical College, Nabari, Japan; h School of Human Science & Environment, University of Hyogo, Himeji, Japan

**Keywords:** Heat transfer coefficient, Infant, Natural convection, Stroller, Thermal environment

## Abstract

Infants have a low capacity to thermally adapt to their environment and so sufficient consideration must be given to their thermal environment. In investigating an infant's thermal environment, the purpose of this study is to clarify the heat transfer coefficient in natural convection for the posture of an infant in a stroller. The heat transfer coefficients were measured by means of using a thermal manikin. The experimental thermal environment conditions were set for eight cases, at: 16 °C, 18 °C, 20 °C, 22 °C, 24 °C, 26 °C, 28 °C, and 30 °C, and the air and wall surface temperatures were equalized, creating a homogeneous thermal environment. The air velocity (less than 0.2 m/s) and relative humidity (50%RH) were the same for each case. The surface temperature of each part of the thermal manikin was controlled to 34 °C. The difference between the mean surface temperature and air temperature (ΔT [K]) is the driving force for the heat transfer coefficient in natural convection for the posture of an infant in a stroller (h_c_ [W/(m^2^·K)]). We propose the use of the empirical formula h_c_ = 2.16 ΔT ^0^^.23^. The formula of the convective heat transfer coefficient in natural convection of this study can be applied to infants up to about 3 years old.

## Highlights

• The formula for the convective heat transfer coefficient of an infant was proposed.
• The experiments were used by means of an infant thermal manikin.
• This heat transfer coefficient can be applied to infants up to about 3 years old.

## Teaser

Heat transfer coefficientL; Infant, Natural convection; Stroller; Thermal environment.

## 1 Introduction

The average global temperature continues to increase, causing previously unknown disasters ranging from extreme winds and torrential rain to the destruction of crops from unusual weather. This presages the possibility of a worsening outdoor thermal environment.

Compared to adults, infants have a low capacity to thermally adapt to the environment. The outdoor environment in summer has become increasingly severe, from a thermoregulation aspect, and so preventative measures must be considered for the thermal environment. The body of an infant characteristically gets warm easily in hot environments and loses heat easily in cold environments (Iriki, 1995). Also, the thickness of an infant's skin is 1/2 to 1/3 of that of an adult (Whitton and Everall, 1973), making an infant's skin temperature higher (Oketani and Tokura, 1979). Therefore, an infant is considered to have a different adaptation to, and sense of, an environment different from an adult (Iriki, 1995; Nakayama and Iriki, 1987). An infant's body surface area is extremely small compared to that of an adult; however, their body surface area per unit body weight is extremely high. Therefore, the heat transfer related to the body temperature regulation is important (Bar-Or, 1980). Besides, infants have less heat transfer involved in thermoregulation than adults (Wagner et al., 1974; Haymes et al., 1975; Drinkwater et al., 1977; Araki et al., 1979; Sugiura et al., 2011). Consequently, the infant has a low capacity to thermally adapt, and require more physical attention (Tsuzuki et al., 1998).

According to the Ministry of the Environment's Heat Illness Prevention Information site (Ministry of the Environment, Government of Ministry of the EnvironmentGovernment of Japan, 2018), when the temperature 1.5 m above the ground is 32 °C in Tokyo, the temperature 0.5 m above ground was shown to be above 35 °C. Half a meter above ground is almost the same height as an infant in a stroller. The effects of reflected solar radiation or thermal radiation and the high surface temperature are thought to be significant. In addition, Tsuchikawa et al. (2015) examined the effects of reflected solar radiation and thermal radiation from the ground surface as concerns the evaluation height from the ground surface. The mean radiant temperature 0.5 m above the ground, which corresponds to the position of an infant in a stroller, is significantly higher than the reflected radiation from the ground at 1.0 m and 1.5 m, which correspond to the chest and head of an adult guardian. Infants are exposed to a more extreme thermal environment than that felt by an adult. Therefore, it could be said that introducing measures based on an adult's sense of heat could expose infants to an increased risk of heatstroke.

To protect infants in strollers from direct sunlight, covering their body with the stroller's sunshade shades them from direct sunlight but prevents circulation of air. Therefore, heat and humidity can easily accumulate in the stroller, increasing the risk of heatstroke. They may thus succumb to so-called stroller heatstroke. Infants have a less developed sense of hydration compared to an adult, and they lose water more quickly and must be rehydrated earlier. However, infants are unable to complain of thirst. Water loss due to an infant's insensible perspiration is 2.5 times that of an adult. Also, water is expelled from the body via urine. An adult's kidneys can filtrate, reabsorb, or expel water, yet an infant's kidneys are not fully developed, so most of their water leaves the body as urine (Maxell and Kleeman, 1972; Hill, 1990; Boineau and Lewy, 1990). Therefore, the infant's water loss is significantly greater, leading to dehydration. In other words, the thermal environment in strollers is conducive to heat stroke.

Thermoregulation in an outdoor environment is affected by thermal sense. In order to begin behavioral thermoregulation to improve a poor thermal environment to a more comfortable one, a thermal sense through a stimulus from the thermal environment is necessary. To prevent degradation of health, it is essential to understand the effects on the body caused by the thermoregulatory load.

As infants cannot complain their own thermal condition, their thermal environment is influenced by the judgment of the parent. However, the body heat balance of an infant has not been evaluated insufficient. As mentioned above, shielding with the stroller's sunshade prevents circulation of air. Therefore, thermal condition can easily accumulate in the stroller, increasing the risk of heatstroke. Consequently, it is essential to make clear the convective heat loss.

Thus, in this study, the heat transfer coefficient in natural convection was clarified in still air, assuming a condition where ventilation is poor and heat and humidity are trapped in the stroller. There is a limiting human factor relating to an infant's thermal environment and the target values are thought to be significant.

## 2 Calculation of convective heat transfer coefficient

The convective heat transfer coefficient h_c_ can be calculated from the sensible heat exchange using the following formulas (1-4).

(1) Q=C+R

(2) $$C=h_{c}\;\left(T_{s}-T_{a}\right)\;f_{conv}$$

(3) $$R=h_{r}\;\left(T_{s}^{4}-T_{r}^{4}\right)\;F_{w-h}\;f_{rad}$$

(4) $$h_{c}=\left(Q-\varepsilon_{h}\;\varepsilon_{w}\;\sigma \;\left(T_{s}^{4}-T_{r}^{4}\right)\;F_{w-h}\;f_{rad}\right)\;/\;\left(\left(T_{s}-T_{a}\right)\;f_{conv}\right)$$

Here,

C: Convective heat exchange [W/m^2^]

f_conv_: Convective heat transfer area factor [N.D.]

f_rad_: Radiant heat transfer area factor [N.D.]

F_w-h_: Angle factor between thermal manikin and wall [N.D.]

h_c_: Convective heat transfer coefficient [W/(m^2^∙K)]

h_r_: Radiant heat transfer coefficient [W/(m^2^∙K)]

Q: Sensible heat exchange [W/m^2^]

R: Radiant heat exchange [W/m^2^]

T_a_: Air temperature [K]

T_r_: Mean radiant temperature [K]

T_s_: Mean surface temperature of thermal manikin [K]

ε_h_: Emissivity of thermal manikin [N.D.]
ε_w_: Emissivity of wall [N.D.].

The radiant heat transfer area factor f_rad_ can be calculated using the following formula (5).

(5) $$f_{\mathrm{rad}}=A_{\mathrm{rad}}\;/\;A_{s}$$

Here,

A_rad_: Radiant heat transfer area [m^2^]

A_s_: Total body surface area of the thermal manikin [m^2^]

f_rad_: Radiant heat transfer area factor [N.D.].

The convective heat transfer area factor f_conv_ can be calculated using the following formula (6).

(6) $$f_{conv}=A_{conv}\;/\;A_{s}$$

Here,

A_conv_: Convective heat transfer area [m^2^]

A_s_: Total body surface area of the thermal manikin [m^2^]

f_conv_: Convective heat transfer area factor [N.D.].

The radiant heat transfer coefficient *h*_*r*_ is calculated by body heat balance. However, studies stating a specific value for it are extremely rare. Stolwijk (1970), Mochida (1977), Tanabe et al. (1994), de Dear et al. (1997), Ichihara et al. (1997), and Kurazumi et al., 2006, 2008a, 2008b, 2013) proposed specific values. Stolwijk (1970), Mochida (1977), de Dear et al. (1997), Ichihara et al. (1997), and Kurazumi et al., 2006, 2008b, 2008c) proposed the values for a human model. Kurazumi et al. (2013) proposed the values for a human body. Tanabe et al. (1994) calculated to using a linear formula (ASHRAE, 2017) for the radiant heat transfer coefficient. , Kurazumi et al., 2006, 2008a, 2008b, 2013) directly measured the radiant heat transfer coefficient. Most of these studies do not measure the radiant heat transfer exchange. They were calculated to use the Stefan‒Boltzmann law by means of the angle factor between each body part and the surface of the space. In the previous studies, the angle factors for the local body were not proposed, and the proposed values are questionable. Moreover, the previous studies did not propose the human factors relating to the thermal environment, such as the radiant heat transfer area or angle factor for an infant body.

Mochida (1977) stated that, in indoor space, radiant heat exchange is mainly in the region of thermal radiation, so general objects can be regarded as gray bodies. In addition, the emissivity of the skin and clothing is close to that of a blackbody, regardless of their color. Therefore, it is possible to treat the emissivity and the temperature coefficient collectively as constants in the temperature range in the daily living area, and treat the radiant heat transfer coefficient of the human body as a constant value. Therefore, in this study, the linear equation (ASHRAE, 2017) for the radiant heat exchange of an infant body was used.

Previous studies have shown that the radiant heat transfer area factor of the human body in a sitting posture similar to a reclining posture ranges from 0.70 to 0.76 (Guibert and Taylor, 1952; Fanger et al., 1970; Horikoshi and Kobayashi, 1982; Kakitsuba et al., 1987; Tsuchikawa et al., 1988, 1991; Horikoshi et al., 1990; Kakitsuba and Suzuki, 1997; Tomita and Miyamoto, 2001; Miyamoto et al., 2003; Kurazumi et al., 2008c). As mentioned above, the value of 0.70 from Fanger et al. (1970), which is used in the linear equation (ASHRAE, 2017), was substituted, since the radiant heat transfer area factor of an infant has not been proposed.

Most of the previous studies have used 1.0 for the convective heat transfer area factor for calculation of the convective heat exchange. However, Kurazumi et al. (2003, 2004, 2008c) stated that 10–20% of the body surface area did not function to the convective heat transfer area even in the standing and seated positions. In this study, 1.0 was used for the convective heat transfer area factor because, as described later, the experimental method is to open the infant thermal manikin, which does not touch the body surface, to the air.

## 3 Experimental plan

Some studies have examined on experimental formulas. Some have considered simple heat emitting devices or assemblies to model the convective heat transfer coefficient (Stolwijk, 1970; Rapp, 1973; Mochida, 1977; Lee et al., 1990; Ohmoto and Mochida, 1995). Some have considered subjects to investigate the body heat balance (Büttner, 1934; Winslow et al., 1936, 1939; Hardy and DuBois, 1938; Nelson et al., 1947; Nielsen and Pedersen, 1952; Colin and Houdas, 1967; Woodcock and Breckenridge, 1965; Mitchell et al., 1969; Clark et al., 1974; Clark and Toy, 1975; Galimide et al., 1979; Ishigaki et al., 1993; Ishii et al., 2000). Some have considered a thermal manikin (Nielsen and Pedersen, 1952; Lee et al., 1991; Tanabe et al., 1994; deDear et al., 1997; Ichihara et al., 1997; Mochida et al., 1999; Kuwabara et al., 2001; Ozeki et al., 2002; Oguro et al., 2002a, 2002b; Kurazumi et al., 2006, 2008a, 2008b, 2013, 2014; Watanabe et al., 2008; Luo et al., 2014, 2015; Li and Ito, 2014; Yang et al., 2018; Yu et al., 2020). Some have considered a numerical model (Zeng et al., 1998; Murakami et al., 1999; Oguro et al., 2002a, 2002b; Ono et al., 2008; Luo et al., 2014, 2015; Li and Ito, 2014; Yang et al., 2018). And others have considered the mass and heat transport properties of naphthalene (Nishi and Gagge, 1970; Neal, 1975; Chang et al., 1988). The convective heat transfer coefficients in these studies are different values depending on experimental methods and calculation theories. Since the shape of the human body is complicated, this depends on whether the measured value of the human body used for calculating the convective heat transfer coefficient is appropriate as a representative value of the whole body or parts. It also depends on whether it is effective as an actual measurement accuracy.

Experiments on test subjects in thermal environments based on the body heat balance for infants are extremely difficult. Therefore, to investigate an infant's thermal environment, the infant's human factor relating to the infant's thermal environment must be clarified and experiments using a human thermal model or simulation should be considered.

Even if the simulation method is used, it is not possible to evaluate whether the results are appropriate or not under the current situation where the human factors of infants are not clear. However, the method of the thermal manikin has the following problems, which should be noted. Since the thermal manikin does not possess a heat loss mechanism, and is only cooled via natural heat loss, it is essential that the thermal environmental conditions are set at the surface temperature of a thermal manikin or less, at which the surface temperature of the thermal manikin could be controlled accurately. In addition, the thermal manikin has no contacted areas between skin surfaces.

Furthermore, in this study, the thermal manikin is only sensible heat dissipation. Kuno (1963) stated that, in a cool environment where a steady state is maintained within the range where heat production due to shivering does not occur, there is no perspiration in heat dissipation from vapor perspiration, and most of it is only insensible perspiration, which is small and constant. Moreover, even in a comfortable state, sweating is almost negligible. Therefore, experiments in a thermal environment where the air temperature is higher than the surface temperature cannot be assumed.

**Figure 1:**
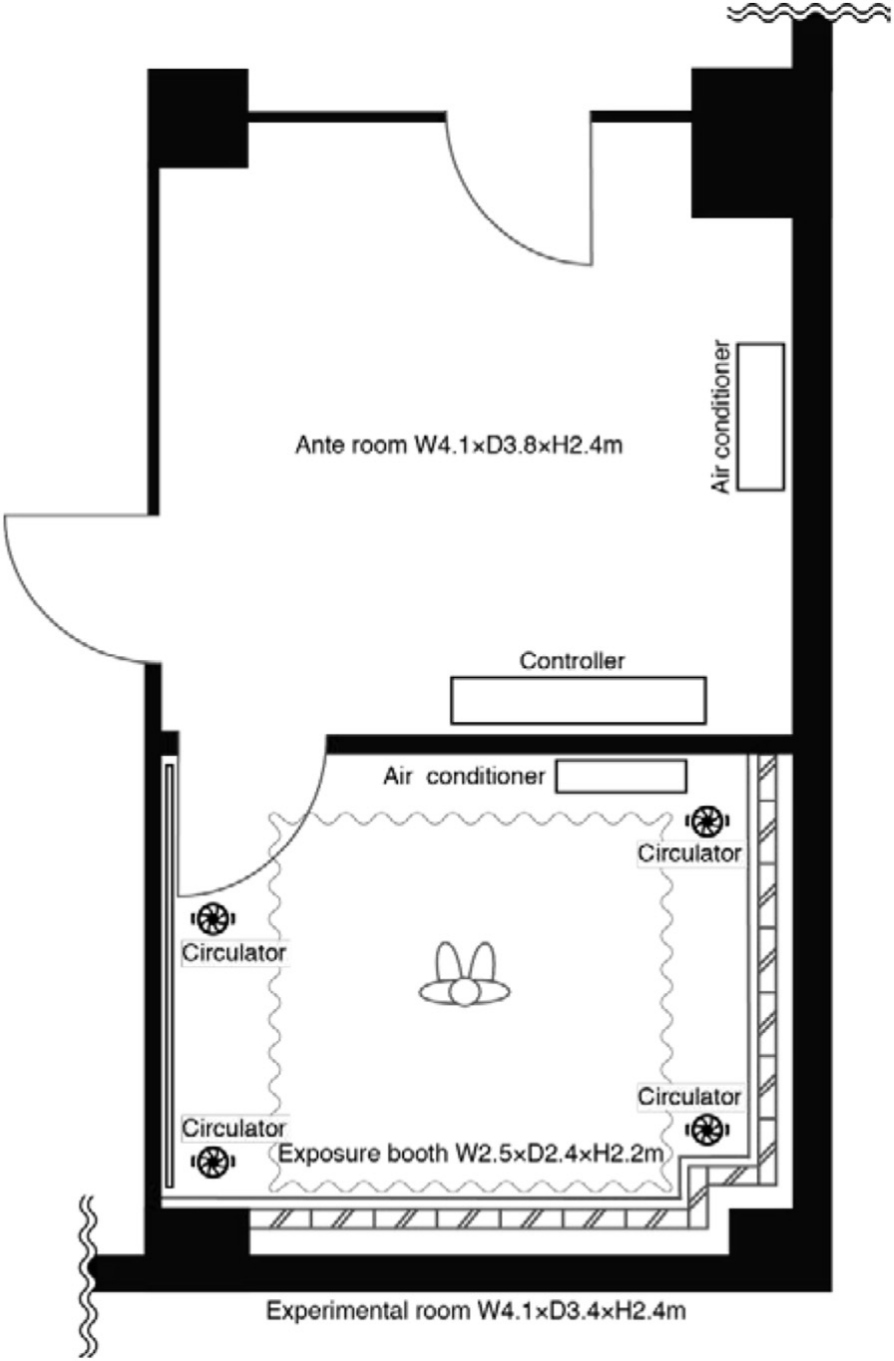
Plan of experimental set up where subjects are exposed to thermal conditions.

The experimental room shown in Figure 1 was used for the experiment. Using an infant for measurements in the experiment is unethical. The experiment therefore used an infant thermal manikin, developed based on the method by Kurazumi et al. (2019). The age of 1–2 months from the Ministry of Health, Labour and Welfare of Japan Survey of Male Infant Development (2021), was found a height of 55.6 cm and weight of 4.79 kg Kurazumi et al. (2019) stated that the validity of this as an infant human model. Table 1 shows the body surface area of the thermal manikin.

**Table 1:** Characteristics of infant thermal manikin.

Region	Surface area [cm^2^]	Area ratio [-]	Area ratio [-]
Anterior head	332.20	0.141	0.227
Posterior head	203.45	0.086	
Ventral trunk	317.36	0.135	0.283
Dorsal trunk	348.34	0.148	
Right arm	132.79	0.056	0.117
Left arm	143.34	0.061	
Right hand	62.08	0.026	0.047
Left hand	50.59	0.021	
Right leg	310.79	0.132	0.258
Left leg	297.75	0.126	
Right foot	82.00	0.035	0.068
Left foot	77.84	0.033	

The thermal manikin manganin heating wire and alumel measurement wire were distributed over the following parts: head, trunk, arm, hand, leg, foot. The thermal manikin had structure, comprising heating layer and temperature measuring layer. The thermal manikin energizes the heating system circuit from the DC power supply, and heats the manganin wire. Furthermore, by measuring the voltage and current flowing through the manganin wire, the calorific value of the manganin wire is calculated sequentially. From the surface temperature measurement system circuit, the surface temperature is calculated. The voltage is controlled by PID control so that the surface temperature of the thermal manikin can be maintained at the set temperature. The surface temperature was controlled to 34 °C.

**Figure 2:**
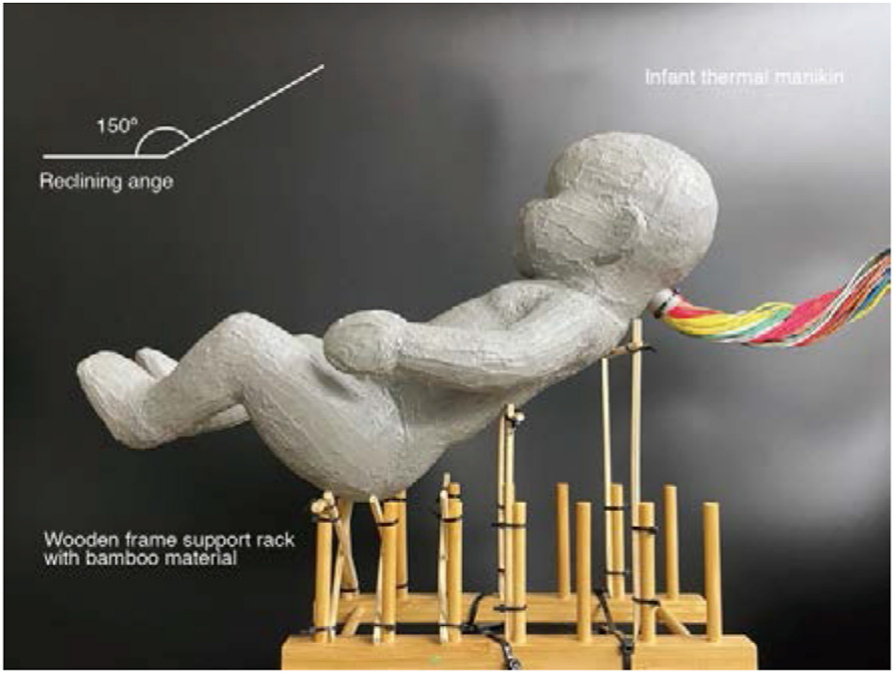
Experimental infant thermal manikin.

Standards for the stroller (British Standards, 1997; SG standard, 2017; European Standards, 2018; ASTM Standards, 2019) are stipulated for safe assembly; however only CPSA001 (SG standard, 2017) was standardized for the stroller reclining angle. The CPSA001 (SG standard, 2017) for an A-type stroller states that, for infants between 1 and 4 months old (who cannot yet hold their head up) or sleeping infants, the stroller must have a reclining angle of 150°. For infants between 4 and 48 months, the stroller must have a reclining angle of over 130°. Therefore, this study utilized a backrest angle of 150° with the infant in a reclined seated position (Figure 2).

The thermal manikin was placed directly on a well-ventilated pedestal made of 5 mm bamboo slats. Since the pedestal has a low heat capacity and the contacted area with the thermal manikin is very small, the manikin is considered to be open to the air.

Table 2 shows the experimental thermal environment conditions. Considering that the heat dissipation control of the thermal manikin is the natural cooling mechanism, the experimental thermal environment conditions were set from 30 °C to the low temperature range where the surface temperature of the thermal manikin can be controlled. The experimental thermal environment conditions were set for eight cases, at: 16 °C, 18 °C, 20 °C, 22 °C, 24 °C, 26 °C, 28 °C, and 30 °C and the air and wall surface temperatures were equalized, creating a homogeneous thermal environment. The air velocity (less than 0.2 m/s) and relative humidity (50%RH) were the same for each case.

**Table 2:** Experimental conditions.

Air Temperature [ºC]:T_a_	Air Velocity [m/s]: V_a_	Relative Humidity [%]: RH	Mean Radiant Temperature [ºC]:MRT
16.0			
18.0			
20.0			
22.0	<0.2	= 50	= Ta
24.0			
26.0			
28.0			
30.0			

The thermal environment conditions were measured the air temperature, humidity, air velocity, and the wall temperatures. The air temperature and humidity were measured with an Assmann ventilated psychrometer, the air velocity was measured with an omnidirectional hot bulb type anemometer, and the walls temperature were measured with a 0.2 mm φ T-type thermocouple.

The surface temperature and heat loss of the thermal manikin were measured. After verifying that the thermal environmental conditions and heat loss from the thermal manikin had reached a steady state, the manikin was exposed to the set thermal environmental conditions for 90 min. The analyzed data was the 60-minute period between 20 min after the start and 10 min before the end of the experiment.

## 4 Results and discussion

Table 3 shows the measurements of the experimental thermal environmental conditions. Although there is an approximately 0.2 °C deviation for each temperature condition, it was controlled to within ±0.1 °C. Although there is an approximately 6% variation in relative humidity, it was controlled to within ±0.8%. There was almost 2.3 °C variation between the surface of the walls and the surface of the floor; however, the temperature was similar. The angle factor of the infant was not proposed. However, the area weighted mean radiant temperature was similar to the air temperature. The air velocity was below 0.2 m/s. The thermal environmental conditions mostly met the set conditions.

**Table 3:** Results of experimental condition.

Air Temperature [ºC]:T_a_	Air Velocity [m/s]: V_a_	Relative Humidity [%]: RH	Floor Temperature [ºC]:T_f_	Wall Temperature [ºC]:T_w_	Ceiling Temperature [ºC]:T_c_	Mean Radiant Temperature [ºC]: MRT
16.1 ± 0.1	0.12 ± 0.04	48.3 ± 0.8	15.7 ± 0.0	16.3 ± 0.1	15.7 ± 0.6	16.1 ± 0.1
17.9 ± 0.1	0.14 ± 0.04	55.8 ± 0.4	17.2 ± 0.1	17.9 ± 0.1	17.9 ± 0.1	17.9 ± 0.1
20.0 ± 0.1	0.13 ± 0.05	55.8 ± 0.4	19.2 ± 0.0	20.1 ± 0.1	20.2 ± 0.1	20.0 ± 0.1
22.2 ± 0.1	0.11 ± 0.04	55.8 ± 0.4	21.0 ± 0.1	22.4 ± 0.1	22.9 ± 0.1	22.2 ± 0.1
24.1 ± 0.1	0.11 ± 0.04	55.5 ± 0.5	22.8 ± 0.0	24.4 ± 0.1	25.1 ± 0.1	24.2 ± 0.1
25.9 ± 0.1	0.11 ± 0.04	55.8 ± 0.4	24.2 ± 0.1	26.2 ± 0.1	27.0 ± 0.1	26.0 ± 0.1
28.1 ± 0.1	0.10 ± 0.04	55.8 ± 0.4	25.9 ± 0.1	28.4 ± 0.1	29.7 ± 0.1	28.2 ± 0.1
30.1 ± 0.1	0.10 ± 0.04	55.5 ± 0.5	27.8 ± 0.1	30.4 ± 0.1	32.3 ± 0.1	30.3 ± 0.1

**Figure 3:**
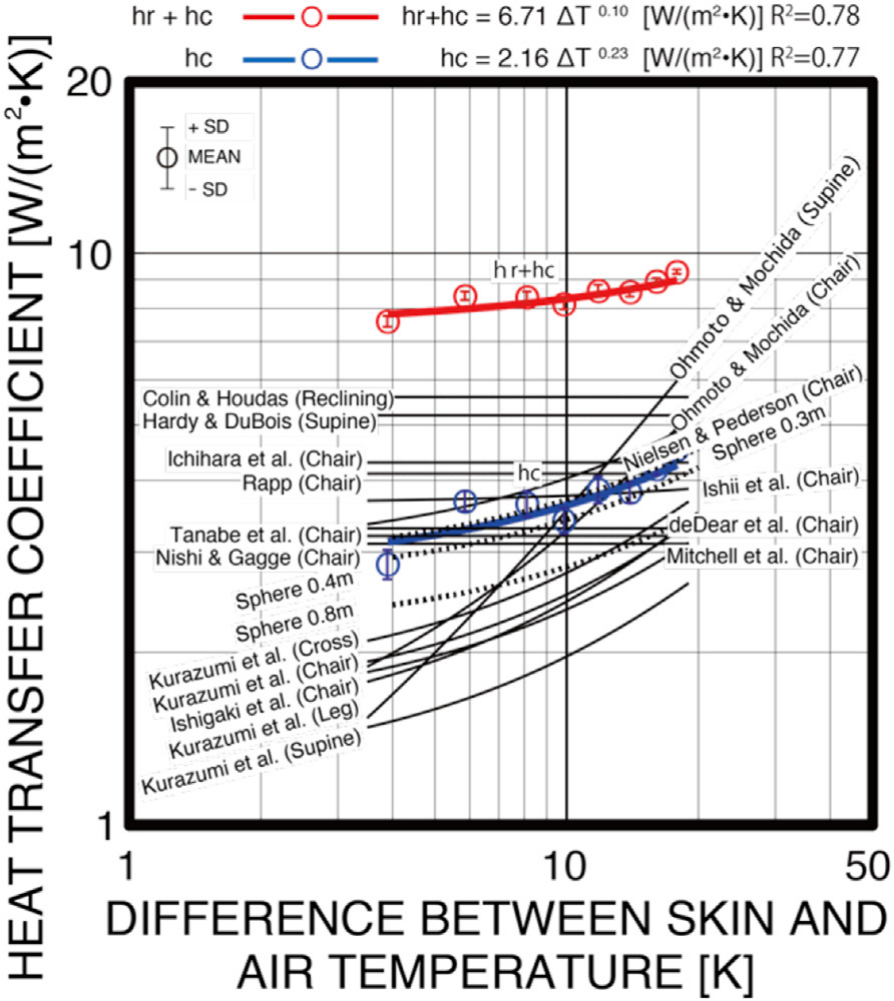
Comparison of convective heat transfer coefficients of the human body in previous studies. Chair is the chair sitting position. Reclining is the reclining chair sitting position. Supine is the supine position. Kurazumi et al. (Chair) is the chair sitting (contact with seat, chair back and floor) position with the area of contact between the body and the chair and the area of contact between the body and the floor. Kurazumi et al. (Cross) is the cross-legged sitting (floor contact) position with the area of contact between the body and the floor. Kurazumi et al. (Leg) is the leg-out sitting (floor contact) position with the area of contact between the body and the floor. Kurazumi et al. (Supine) is the supine (floor contact) position with the area of contact between the body and the floor.

Figure 3 shows the heat transfer coefficient in natural convection of an infant, calculated from the body heat loss and the operative temperature. This study only handled the sensible heat exchange. In addition, since the pedestal used to maintain posture has a low heat capacity and the contacted area with the thermal manikin is small, the thermal manikin is considered to be open to the air. Therefore, the heat exchange paths are only radiant and convective heat exchange. The difference between the air temperature of the thermal environment's set conditions and the surface temperature of the infant was within the range of 4–18 °C, and the heat transfer coefficient was about 8–9 W/(m^2^•K). From the results of testing the significance of the regression coefficients in a regression equation by ANOVA, where p < 0.05 (p = 0.00), a significant regression trend was effectively determined. The difference (ΔT [K]) between the mean skin temperature *T*_*s*_ and the air temperature *T*_*a*_ is the driving force for the overall heat transfer coefficient (h_r_ + h_c_ [W/(m^2^•K)]) in natural convection and is given by the following regression formula (7):

(7) $$h_{r}+h_{c}=6.71\;{\Delta T}^{0.10}\left[W/\left(m^{2}•K\right)\right]$$

Here,

h_r_: Radiant heat transfer coefficient [W/(m^2^•K)]

h_c_: Convective heat transfer coefficient [W/(m^2^•K)]

ΔT: T_s_-T_a_ [K]

T_s_: Mean skin temperature [K]

T_a_: Air temperature [K].

Figure 3 shows a comparison of the convective heat transfer coefficient of an infant with previous studies. From the results of testing the significance of the regression coefficients in a regression equation by ANOVA, where p < 0.05 (p = 0.00), a significant regression trend was effectively determined. The difference (ΔT [K]) between the mean skin temperature *T*_*s*_ and the air temperature *T*_*a*_ is the driving force for the convective heat transfer coefficient (h_c_ [W/(m^2^•K)]) in natural convection and is given by the following regression formula (8):

(8) $$h_{c}=2.16\;{\Delta T}^{0.23}\left[W/\left(m^{2}•K\right)\right]$$

Here,

h_c_: Convective heat transfer coefficient [W/(m^2^•K)]

ΔT: T_s_-T_a_ [K]

T_s_: Mean skin temperature [K]

T_a_: Air temperature [K].

The coefficients of determination of the regression formulas for the overall heat transfer coefficient (h_r_ + h_c_) and the convective heat transfer coefficient (h_c_) are 0.78 and 0.77, respectively, showing a strong correlation.

As mentioned in the calculation of the radiant heat exchange, since the infant factors related to the thermal environment such as the radiant heat transfer area and the angle factor of the infant body have not been clarified, in this study, the linear equation (ASHRAE, 2017) for the radiant heat exchange of an infant body was used. Therefore, in reality, the radiant heat exchange is expected to be less than that calculated by the linear equation (ASHRAE, 2017). Moreover, in this study, the convective heat transfer area factor is 1.0, because the body surface is completely open to the air. Kurazumi et al. (2003, 2004, 2008c) stated that 10–20% of the body surface area did not function to the convective heat transfer area even in the standing and seated positions. Therefore, in reality, the convective heat exchange is expected to be less than this. It is unclear whether these factors cancel each other out, and it is necessary to examine this based on progress in research on thermal coefficient factors in infants.

The previous study by Kurazumi et al. (2008a) included considerations of the convective heat transfer area in a chair sitting position, cross-legged sitting position, leg-out sitting position, and supine position. This showed a low value when compared with the convective heat transfer coefficient open to the air (Kurazumi et al., 2008a). Colin and Houdas (1967) in the reclining position and Hardy and DuBois (1938) in the supine position show higher convective heat transfer coefficients than the other studies of the sitting position. Kurazumi et al. (2008a) show lower convective heat transfer coefficient than the other studies. Kurazumi et al. (2008a) take into account the convective heat transfer area factor, but the other studies assume the convective heat transfer area factor to be 1.0. It can be said that the convective heat transfer area factor strongly affects the convective heat transfer coefficient. The convective heat transfer coefficient of a reclining infant could be inferred to be within the range of a seated body and a supine body fully open to the air. The reclining position of a body fully open to the air as studied by Colin and Houdas (1967) involved a chair sitting position on a shallow seat, but there was no mention of a specific reclining angle. The convective heat transfer coefficient in natural convection is assumed to be independent of the difference between air temperature and mean skin temperature. Similarly, studies by Hardy and DuBois (1938), Ichihara et al. (1997), Rapp (1973), deDear et al. (1997), Tanabe and Hasebe (1993), Nishi and Gagge (1970), and Mitchell et al. (1969) all assumed to be independent of the difference between air temperature and mean skin temperature. Each study had different set air temperatures, yet they were all conducted under still air flow conditions.

Fujii and Imura (1972) and the JSME Data Book (The Japan Society of Mechanical Engineers, 1986) stated that the convective heat transfer coefficient in natural convection tends to decrease as the inclination angle of the heated object increases. These results are consistent with this study.

However, studies by Ohmoto and Mochida (1995), Nielsen and Pedersen (1952), Ishii et al. (2000), Ishigaki et al. (1993), and Kurazumi et al. (2008a, b, c) had the difference between the air temperature and mean skin temperature as the driving force. Studies by Nielsen and Pedersen (1952), Ishigaki et al. (1993), and Kurazumi et al. (2008a) tended to have a similar dependency on the difference between the air temperature and mean skin temperature. The convective heat transfer coefficient of an infant was almost equal to that found by Nielsen and Pedersen (1952).

There are various postures and physiques of people, and it is natural that the value of the convective heat transfer coefficients differs according to the state. Therefore, the convective heat transfer coefficient needs to be considered individually, depending on the posture and physiques. As mentioned above, in this study, the infant is about 1–2 months old. In the Ministry of Health, Labour and Welfare of Japan Survey of Male Infant Development (2021), the age of 1–2 months was found a height of 55.6 cm and weight of 4.79 kg. The age of 12 months was found a height of 74.8 cm and weight of 9.24 kg. The age of 24 months was found a height of 86.7 cm and weight of 11.93 kg. The age of 36 months was found a height of 95.1 cm and weight of 13.99 kg.

An infant riding in a stroller assumes a reclining posture, and can be expressed as a man-equivalent thermal sphere. When the body surface area of an infant is calculated by Fujimoto et al. (1968) from the above height and weight, the diameters of the man-equivalent thermal spheres are 0.31 m in 1–2 months, 0.38 m in 12 months, 0.41 m in 24 months, and 0.43 m in 36 months, respectively. ASHRAE (2017) stated the body surface area of a typical average adult at 1.83 m^2^. In the same way as for infants, calculating the diameter of the man-equivalent thermal sphere in the chair sitting position, it is 0.76 m. Considering the age of infants riding in strollers, the diameter of the man-equivalent thermal sphere is considered to be about 0.3–0.4 m. In addition to consider the adult of chair sitting position, the diameter of the man-equivalent thermal sphere is considered to be about 0.8 m. A man-equivalent thermal solid is a simple solid whose body surface area or volume is the same as that of the human body, which has a complicated shape. In this study, the man-equivalent thermal solid was assumed to have the same body surface area in order to make the heat transfer area equivalent.

Assuming that the sitting position is equivalent to a sphere, the natural convective heat transfer can be expressed as a function of the Rayleigh number Ra. Therefore, the representative length affects the Grashof number Gr and Prandtl number Pr (The Japan Society of Mechanical Engineers, 1986). Therefore, the representative length affects the Grashof number Gr and Prandtl number Pr. The results of the Ranz-Marshall formula are showed Figure 3. As shown in Table 1, the thermal manikin in this study has a body surface area of 0.24 m^2^. Calculating the diameter of the man-equivalent thermal sphere is 0.28 m. The results of this study and the results of calculating the convective heat transfer coefficient by the Ranz-Marshall formula with the diameter of the man-equivalent thermal sphere 0.3 m are almost consistent.

An inspection of the parallelism of the regression lines gave p < 0.01 (RMSE = 0.13, F = 7.20, p = 0.00), indicating a significant difference in the parallelism of the regression lines. An inspection of the homogeneity of regression gave p < 0.01 (RMSE = 0.03, F = 113.62, p = 0.00), indicating a significant difference in the homogeneity of the regression lines. As a result of multiple comparison by Tukey-Kramer, measured result and infants (diameter of man-equivalent thermal sphere 0.3 m, 0.4 m) were p > 0.01, which do not indicate a significant difference. However, the diameter of the man-equivalent thermal sphere 0.8 m (adult) and the infants (diameter of man-equivalent thermal sphere 0.3 m, 0.4 m, measured result) were p < 0.01, which indicated a significant difference. It can be considered that the convective heat transfer coefficients of the adults and the infants are different. Considering the above hypothetical calculation of the natural convective heat transfer coefficients, it is considered possible to adapt to the infant in stroller up to about 3 years old.

As mentioned above, studies using the difference between the air temperature and mean skin temperature as the driving force (Ohmoto and Mochida, 1995; Nielsen and Pedersen, 1952; Ishii et al., 2000; Ishigaki et al., 1993; Kurazumi et al., 2008a, b, c) compared with these values are almost the same or higher. As also mentioned above, this result is generally consistent with the calculation results assumed for the sphere.

## 5 Conclusion

Comparing an adult with an infant in a stroller, the effect of thermal radiation from the ground is significantly stronger, raising concerns for thermal illnesses, such as heatstroke. Infants can succumb to so-called ‘stroller heatstroke’ because they have a low capacity to thermally adapt to the environment, and so sufficient consideration must be given to the thermal environment. This study aimed to propose the heretofore unclarified convective heat transfer coefficient in natural convection of an infant.

The empirical formula for the convective heat transfer coefficient (hc [W/(m^2^·K)]) in natural convection of an infant in a stroller with the driving force as the difference (ΔT [K]) between the mean skin temperature and air temperature was proposed as h_c_ = 2.16 ΔT ^0.23^ [W/(m^2^·K)]. The formula of the convective heat transfer coefficient in natural convection of this study can be applied to infants up to about 3 years old.

## Declarations

### Author contribution statement

Yoshihito Kurazumi: Conceived and designed the experiments; Performed the experiments; Analyzed and interpreted the data; Wrote the paper.

Kenta Fukagawa: Performed the experiments; Contributed reagents, materials, analysis tools or data; Wrote the paper.

Tomonori Sakoi: Conceived and designed the experiments; Analyzed and interpreted the data; Wrote the paper.

Ken Yamashita: Analyzed and interpreted the data; Contributed reagents, materials, analysis tools or data; Wrote the paper.

Akie Naito, Motoe Imai, Yoshiaki Yamato, Emi Kondo, Tadahiro Tsuchikawa: Analyzed and interpreted the data; Wrote the paper.

### Funding statement

Associate Professor Tomonori Sakoi was supported by Japan Society for the Promotion of Science KAKENHI Grant Number JP18H01594.

### Data availability statement

Data included in article/supp. material/referenced in article.

### Declaration of interest's statement

The authors declare no conflict of interest.

### Additional information

No additional information is available for this paper.
